# A Continuous-Flow Model for *in vitro* Cultivation of Mixed Microbial Populations Associated With Cystic Fibrosis Airway Infections

**DOI:** 10.3389/fmicb.2019.02713

**Published:** 2019-11-22

**Authors:** Thomas James O’Brien, Martin Welch

**Affiliations:** Department of Biochemistry, University of Cambridge, Cambridge, United Kingdom

**Keywords:** cystic fibrosis, continuous-flow, co-culture, *in vitro*, *Pseudomonas aeruginosa*, *Staphylococcus aureus*, *Candida albicans*

## Abstract

The airways of people with cystic fibrosis (CF) provide a nutrient-rich environment which favours colonisation by a variety of bacteria and fungi. Although the dominant pathogen associated with CF airway infections is *Pseudomonas aeruginosa*, it is becoming increasingly clear that inter-species interactions between *P. aeruginosa* and other colonists in the airways may have a large impact on microbial physiology and virulence. However, there are currently no suitable experimental models that permit long-term co-culture of *P. aeruginosa* with other CF-associated pathogens. Here, we redress this problem by describing a “3R’s-compliant” continuous-flow *in vitro* culture model which enables long-term co-culture of three representative CF-associated microbes: *P. aeruginosa*, *Staphylococcus aureus* and *Candida albicans*. Although these species rapidly out-compete one another when grown together or in pairs in batch culture, we show that in a continuously-fed setup, they can be maintained in a very stable, steady-state community. We use our system to show that even numerically (0.1%) minor species can have a major impact on intercellular signalling by *P. aeruginosa*. Importantly, we also show that co-culturing does not appear to influence species mutation rates, further reinforcing the notion that the system favours stability rather than divergence. The model is experimentally tractable and offers an inexpensive yet robust means of investigating inter-species interactions between CF pathogens.

## Introduction

Cystic fibrosis (CF) is the most common life-limiting genetic disorder within the Caucasian population (Cystic Fibrosis Foundation, 2019), with 1 in 40 people estimated to carry the common ΔF508 mutation in the CF transmembrane conductance regulator (CFTR) gene (Bobadilla et al., 2002; Cystic Fibrosis Mutation Database, 2019). The most striking consequence of dysfunctional CFTR activity is the overproduction of nutrient-rich, mucilaginous sputum. This blocks the airways and generates a heterogenous environment with steep oxygen gradients and a lowered pH (Boucher, 2002; Tate et al., 2002; Worlitzsch et al., 2002). This environmental niche is rich in nutrients such as mucin, amino acids, iron, and nitrate making the CF airway prone to colonisation by a variety of microbial species (Grasemann et al., 1998; Jones et al., 2000; Palmer et al., 2007; Ghio et al., 2013). The resulting infections often persist for decades, leading to respiratory failure and eventually, premature death (Lyczak et al., 2002; Rajan and Saiman, 2002; Carmody et al., 2013, 2015; Elborn, 2016). Traditionally, these CF-associated infections have been linked with a relatively small number of easily-culturable pathogens, such as *Pseudomonas aeruginosa* or *Staphylococcus aureus*. However, the introduction of culture-independent molecular profiling approaches revealed that expectorated CF sputum samples often contain a much wider range of bacterial and fungal species (Sibley et al., 2006, 2008; Rogers et al., 2010a; Zhao et al., 2012; Carmody et al., 2013, 2015; Short et al., 2014; Boutin et al., 2015). This suggests that the CF airways may harbour a highly-diverse microbial community, although this notion has been challenged recently through the direct sampling of lavage fluid from the lungs of CF children. These new data suggest that to a large extent, the diversity of the previously reported CF-associated microbiome arises from contamination of the sample during passage through the oral cavity, and following sample processing (Jorth et al., 2019). Nevertheless, these newer studies still suggest that the CF airways harbour a core population of “non-conventional” pathogens, including *Prevotella*, *Veillonella* and *Staphylococcus* species, as well as “traditional CF pathogens” such as *P. aeruginosa* (PA).

The polymicrobial character of many CF-associated airway infections makes it crucial to consider what impact inter-species interactions have on the physiology and composition of the microbial consortium. Previous reports demonstrate that co-culturing bacterial species *in vitro* and *in vivo* causes significant alterations in gene essentiality (Ibberson et al., 2017). This can lead to changes in microbial lifestyle, impacting upon the expression of virulence factors (Rogers et al., 2009, 2010a,b; Hibbing et al., 2010; Leekha et al., 2011; Elias and Banin, 2012; Quinn et al., 2014; Limoli et al., 2016). For example, PA senses peptidoglycan shed from Gram-positive bacteria, and this stimulates the production of extracellular lytic virulence factors (Korgaonkar et al., 2013). Polymicrobial communities also display altered responses to therapeutic intervention. This may explain why many of the currently used clinical interventions designed to target PA show varying degrees of efficacy between patients (Lopes et al., 2012; Peters et al., 2012). As a consequence of these conceptual realisations, research focus is gradually moving away from studying individual species in isolation toward co-cultivating the major CF associated pathogens (Spasenovski et al., 2010; Bragonzi et al., 2012; Filkins et al., 2015; Magalhães et al., 2016; Lopes et al., 2017; Makovcova et al., 2017). However, these efforts are hampered by the paucity of adequate polymicrobial infection models. The development of a model which enables the stable and long-term recapitulation of CF polymicrobial communities is therefore highly-desirable.

**FIGURE 1:**
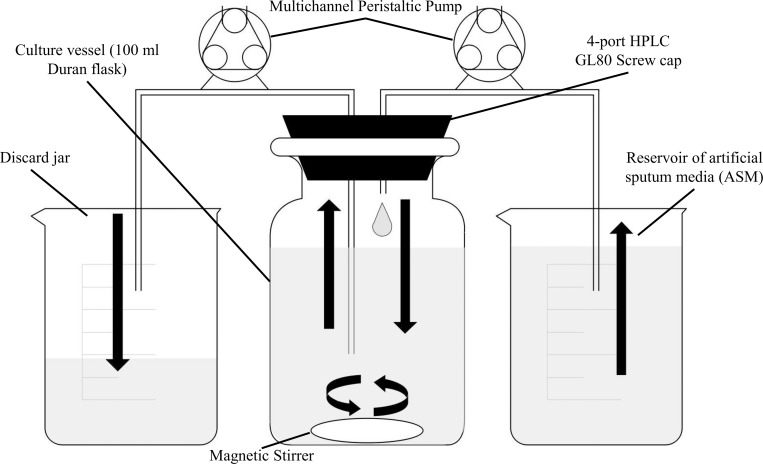
Schematic diagram of the *in vitro* continuous-flow culture system used in this study. The main culture vessel (centre) is a 100 mL Duran bottle fitted with a 4-port HPLC GL80 screwcap lid, containing 4 sealable inlet/outlet ports. A multichannel peristaltic pump delivers fresh media (ASM) into the culture vessel from a reservoir, and also removes waste culture at the same rate of flow (Q). Arrows show the direction of flow. The culture vessel is incubated at 37°C and the contents are kept homogenous through gentle stirring. If required, an in-line spectrophotometer fitted with a continuous-flow cuvette can be included in the setup prior to the discard jar. The value for Q depends on the microbial species being cultured within the vessel.

Here we describe the development of a simple *in vitro* continuous-flow co-culture model which utilises artificial sputum medium (ASM). ASM is known to physiologically recapitulate the nutritional composition of CF airway secretions (Palmer et al., 2007; Kirchner et al., 2012; Turner et al., 2015). To the best of our knowledge, our co-culture model (Figure 1) is the first to permit the long-term, steady-state co-culture of three distinct microbial species: PA, *Staphylococcus aureus* (SA), and *Candida albicans* (CA). Through viable cell counting and optical density measurements, we demonstrate that the abundance of each member of this microbial population remains unchanged over the course of 4 days and that a total carrying capacity can be reached and maintained within the culture vessel. By contrast, and in line with previous reports, when these species are co-cultured under batch conditions, PA rapidly outcompetes the other species (Machan et al., 1992; Duan et al., 2003; Hogan et al., 2004; Mashburn et al., 2005; McAlester et al., 2008; Cugini et al., 2010; Holcombe et al., 2010; Morales et al., 2010; Park et al., 2012; Korgaonkar et al., 2013; Baldan et al., 2014; Fugère et al., 2014; Rüger et al., 2014; Barnabie and Whiteley, 2015; Filkins et al., 2015; Nguyen et al., 2015; Zago et al., 2015; Nguyen and Oglesby-Sherrouse, 2016). Our *in vitro* model provides a defined and experimentally tractable system which can be used to dissect interspecies interactions and determine the long-term impact of co-cultivation on the physiology and gene expression profiles of CF-associated pathogens. Our *in vitro* model also provides a robust and cheaper alternative to existing *in vivo* infection models, making it compliant with the current trend toward the refinement, replacement and reduction (3Rs) of animal models in research.

The species chosen for inoculation into our co-culture model represent three distinct classes of microorganisms; a Gram-negative species and dominant CF-associated pathogen (PA), a Gram-positive species, often associated with CF airway infections (SA) and a dimorphic fungus (CA), also commonly found in CF airway secretions (Conrad et al., 2013). As such, our continuous-flow model lays a solid groundwork for the development and optimisation of an *in vitro* co-culture model which can be directly inoculated with expectorated CF sputum. This will hopefully enable, in the longer-term, full recapitulation of the CF-associated polymicrobial community in defined laboratory conditions.

## Materials and Methods

### Microbial Strains and Culture Conditions

The bacterial/fungal strains used in this study are shown in Table 1. All bacterial strains were routinely cultured in lysogeny broth (LB) (Formedium) with vigorous aeration at 37°C overnight. Where necessary, cultures were supplemented with 50 μg mL^–1^ carbenicillin (to maintain pSB1057 in the *N*-(3-Oxododecanoyl)-L-homoserine lactone OdDHL biosensor strain) or 10 μg mL^–1^ tetracycline (to maintain pSB536 in the *N*-butanoyl-L-homoserine lactone BHL biosensor strain).

**TABLE 1:** Microbial strains used in this study.

**Strain**	**Description**	**References**
PAO1	*Pseudomonas aeruginosa*, spontaneous chloramphenicol-resistant derivative. Used worldwide as a laboratory reference strain (isolated Melbourne, 1954).	Holloway, 1955
ATCC 25923	*Staphylococcus aureus* Rosenbach (ATCC^®^ 25923D-5^TM^), methicillin sensitive clinical isolate. Laboratory reference strain lacking recombinases and *mecA* (isolated Seattle, 1945).	Treangen et al., 2014
SC5314	*Candida albicans*, clinical isolate commonly used as a wild-type laboratory reference strain (isolated New York, 1980s).	Gillum et al., 1984
PAO1 Δ*pqsA* CTX-*lux*::*pqsA*	PQS biosensor strain. Δ*pqsA* mutant of PAO1 containing a *pqsA* promoter:: *luxCDABE* fusion integrated at a neutral site in the chromosome.	Fletcher et al., 2007
JM109 (pSB1057)	OdDHL biosensor strain. *Escherichia coli* JM109 containing pSB1057.	Winson et al., 1998
JM109 (pSB536)	BHL biosensor strain. *Escherichia coli* JM109 containing pSB536.	Winson et al., 1998

Artificial sputum medium was used for every mono-, dual-, and triple-species cultivation. ASM was made using a modified version of the recipe published by Turner et al. (2015). Briefly, bovine maxillary mucin was replaced with 1.25 g L^–1^ porcine stomach mucin type-II (Sigma-Aldrich) and salmon sperm DNA was replaced with 1 g L^–1^ fish sperm DNA (Sigma-Aldrich) as described by Kirchner et al. (2012). A detailed protocol for preparing ASM can be found in the Supplementary Information (SI.1).

#### Continuous-Flow Culture Vessel and Culture Conditions

A schematic of the continuous-flow culture system is shown in Figure 1. The culture vessel consists of a 100 mL flask (Duran), fitted with an assembled 4-port HPLC GL80 screw cap (Duran). A 24-channel IPC ISM934C standard-speed digital peristaltic pump (Ismatec) was used to deliver sterile ASM at a defined flow rate (Q) through 1.5 mm bore sterilin silicon tubing (Fisher Scientific) to the culture vessel. A different channel on the same pump was used to remove waste culture into a discard jar at the same flow-rate. The culture vessel was maintained at 37°C and its contents were kept homogenous by stirring (100 rpm) using a magnetic stir bar. When necessary, the culture optical density was monitored at 600 nm (OD_600 nm_) by passing the removed waste culture through an in-line 6715 UV series spectrophotometer (Jenway) fitted with a continuous-flow cuvette.

Overnight cultures were washed three times in sterile 1 × phosphate buffered saline (PBS, Oxoid) prior to inoculating the culture vessel. Pre-warmed ASM (100 mL) in the culture vessels was inoculated with the required combination of microbial species. Each species was introduced into the culture vessel to achieve a starting OD_600 nm_ of 0.05. The vessel was incubated for 3 h prior to staring the flow of medium. For mono-species and co-culture experiments not containing CA, the flow rate (Q) was set at 170 μL min^–1^. For co-culture experiments including CA, Q was decreased to 145 μL min^–1^. For all continuous-flow experiments, samples (1 mL volume) for cell enumeration were withdrawn using a syringe fitted with a sterile needle inserted through the rubber septa in the HPLC ports.

#### Aerobic and Stirred Batch Culture Conditions

For aerobic batch cultures, 250 mL Erlenmeyer flasks containing pre-warmed ASM (inoculated with the indicated strains to a starting OD_600 nm_ of 0.05) were incubated at 37°C with vigorous shaking (180 rpm). Stirred batch cultures were set up as described for the continuous-flow experiments (see section “Continuous-Flow Culture Vessel and Culture Conditions”), except with Q = 0 μL min^–1^. For both types of batch culture, samples (1 mL volume) were taken from the culture vessel for OD_600 nm_ analysis and viable cell counting.

### Microbial CFU mL^–1^ Enumeration

Colony forming units (CFU) per mL of culture were determined using the single plate-serial dilution spotting (SP-SDS), as described previously (Thomas et al., 2015). Serial dilutions were made in sterile PBS and 20 μL of each dilution was spotted onto the appropriate selective agar. PA was isolated using pseudomonas agar base (Oxoid) supplemented with cetrimide (200 μg mL^–1^) and sodium nalidixate (15 μg mL^–1^). SA was isolated on mannitol salt agar (Oxoid). CA was isolated on BiGGY agar (Oxoid). During co-culture experiments involving CA, the agar plates used to isolate PA and SA were further supplemented with 5 μg mL^–1^ itraconazole to inhibit the growth of CA. All plates were incubated at 37°C. Pseudomonas agar base and mannitol salt plates were incubated overnight (16 h). BiGGY agar plates were incubated for 24 h. CFU mL^–1^ counts are averages taken from three technical repeats. There was no significant difference between total CFU mL^–1^ counts of pure microbial cultures plated onto either non-selective (LB-agar) or any of the selective agar (*data not shown*).

### Quantification of Quorum Sensing Molecules

Aliquots (1.5 mL) of culture were collected after 24 and 96 h (as indicated) of incubation. The cells were pelleted by centrifugation (15,000 × *g*, 5 min, 20°C) and the supernatant was filtered (0.22 μm pore size). Aliquots of the supernatant were snap frozen in liquid N_2_ and stored at −20°C until use. OdDHL was detected using JM109 (pSB1057). BHL was detected using JM109 (pSB536). PQS was detected using PAO1 Δ*pqsA* CTX-*lux*::*pqsA*. Overnight starter cultures of the reporter strains were sub-cultured in LB supplemented with the appropriate antibiotics and grown to OD_600 nm_ = 1.0. Following this, aliquots (60 μL volume) of the normalised cell culture were transferred to a sterile clear-bottomed black opaque 96-well plate (Greiner Bio-One) containing an equal volume of thawed culture supernatant. The plates were incubated at 30°C with shaking (100 rpm) for 3 h. Bioluminescence was recorded using a FLOUstar Omega plate reader (BMG). Standard curves to calibrate the biosensor outputs were constructed using known concentrations of synthetic quorum sensing molecules dissolved in ASM.

### Quantification of Pyocyanin

Pyocyanin quantification was performed following chloroform extraction of the pigment (Knight et al., 1979). Aliquots (10 mL volume) of culture were collected after 96 h growth and the cells were pelleted (4000 × *g*, 30 min, 4°C). The culture supernatants were filter sterilised (0.22 μm pore size). Chloroform (4.5 mL) was added to 7.5 mL of the cell-free culture supernatant and the suspension was vigorously vortexed for 30 s. The immiscible layers were separated by centrifugation (4000 × *g*, 10 min, 4°C). An aliquot (3 mL volume) of the blue-green chloroform phase was removed and mixed with 1.5 mL 0.2M HCl. The immiscible layers were then separated by centrifugation and 1 mL of the rose-pink phase was transferred to a cuvette. The pyocyanin absorbance was measured at 520 nm using a BioSpectrometer Kinetic spectrophotometer (Eppendorf) and converted to concentration (μg mL^–1^) by multiplying the A_520 nm_ value by 26.6.

### Estimation of Mutation Rates

Mutation rates in the chemostat were measured as described by Foster (2006). Initially, the rate constant (λ) associated with exponential growth of PA and SA co-cultured in ASM, was determined by enumeration of CFU mL^–1^ on selective agar plates. Next, the total cell count (*N*) within the culture vessel at steady-state growth was determined. The number of spontaneous rifampicin resistant PA or SA mutants, *r*, was measured after t_1_ = 0 h, t_2_ = 24 h and t_3_ = 96 h of incubation. Total cell numbers in the chemostat did not change appreciably between 24 and 96 h. The value of *r* was determined by plating aliquots (100 μL volume) of culture onto either pseudomonas isolation agar supplemented with 60 μg mL^–1^ rifampicin (for PA), or mannitol salt agar supplemented with 0.05 μg mL^–1^ rifampicin (for SA). The mutation rate per cell per generation (m) was calculated according to Eq. 2 in Foster (2006);

$$\mu =\frac{1}{N\,\lambda }\,\frac{\left(r_{2}-r_{1}\right)}{\left(t_{2}-t_{1}\right)}$$

### Statistical Analysis

Unless otherwise stated, all data represent the mean ± SD of three independent biological experiments. Results were analysed by one-way or two-way ANOVA (as indicated), or Student’s unpaired *t*-test using GraphPad Prism version 8.2.0, with *P* < 0.05 being considered statistically significant.

## Results

### Mono-Species Continuous-Flow Culture (PAO1)

As a first step, we confirmed that PA, SA and CA could all grow in ASM. This was done by inoculating each species into flat-bottomed microtitre plates containing ASM. The plates were incubated at 37°C with vigorous shaking (180 rpm) in a FluoStar Omega plate reader, and the culture optical density (OD_600_) was monitored every 15 min. PA, SA and CA grew rapidly in ASM, achieving a final OD_600_ of > 1 after 24 h in all cases (Supplementary Figure S1).

**FIGURE 2:**
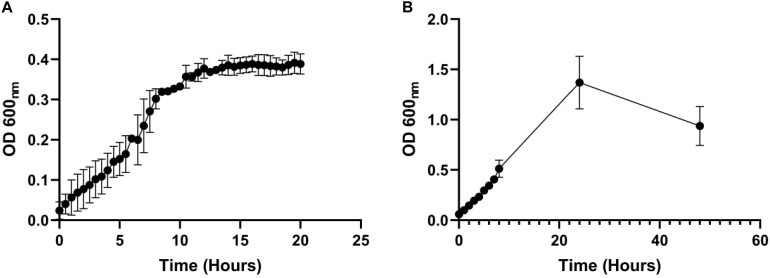
Growth of *P. aeruginosa* in ASM in batch and continuous flow culture conditions. Growth (monitored as OD_600_) of PA in ASM during **(A)** continuous-flow culture (Q = 170 μL min^– 1^); **(B)** batch culture (Q = 0 μL min^– 1^). Data represent the mean ± standard deviation from three independent experiments.

Next, we measured whether a mono-species culture of PA could be maintained with stable steady-state titres in our continuous-flow setup. The laboratory reference strain, PAO1, was inoculated into the continuous-flow system using a flow-rate Q = 170 μL min^–1^ and the OD_600_ was measured every 30 min as described in Section “Continuous-Flow Culture Vessel and Culture Conditions.” The OD_600_ increased almost linearly for the first 8 h and then reached a plateau (OD_600_ ≈ 0.4) after 10 h of incubation (Figure 2A). By comparison, during growth in the same medium and experimental setup with Q = 0 μL min^–1^ (i.e., in stirred batch mode), the PA culture reached a final OD_600_ of > 1 (Figure 2B). We conclude that during continuous-flow operation, the setup allows the culture to achieve a steady-state carrying capacity with an OD_600_ well-below the final OD_600_ associated with entry into the stationary phase of growth in the same medium.

#### Dual-Species Co-culture (PA-SA)

**FIGURE 3:**
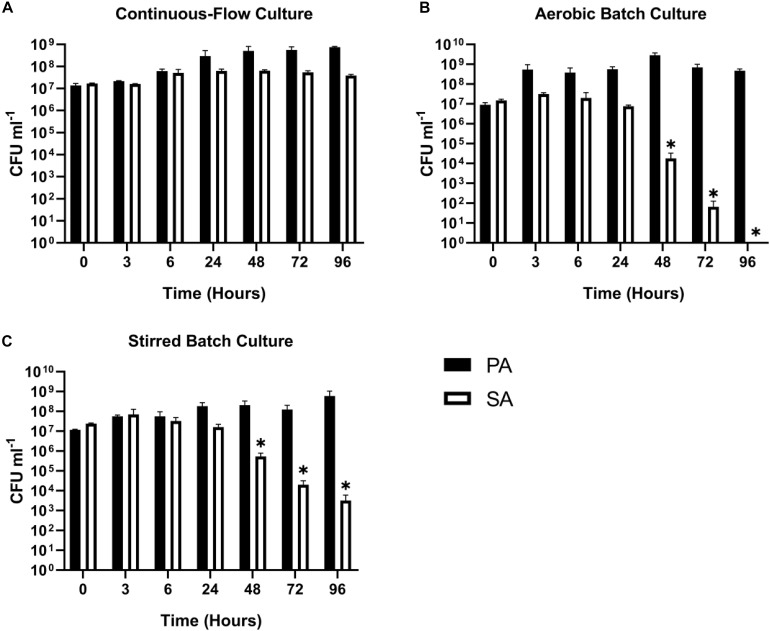
Continuous-flow culture allows *P. aeruginosa* and *S. aureus* to be maintained in a stable steady-state. Viable cell counts [colony forming units (CFUs)] of *P. aeruginosa* PAO1 (PA, black bars) and *S. aureus* 25923 (SA, white bars) during co-culture in ASM using: **(A)** a continuous-flow setup; **(B)** aerobic batch culture; and **(C)** stirred batch culture. Data represent as mean ± standard deviation from three independent experiments. Asterisks represent significant (^∗^*P* < 0.05) differences in CFU mL^– 1^ counts in comparison with the data from the 24 h time point.

*Staphylococcus aureus* is also associated with CF airway infection and is particularly prevalent in adolescent patients (Goss and Muhlebach, 2011; Conrad et al., 2013; Jorth et al., 2019). Despite PA and SA being frequently co-isolated from CF patients, numerous antagonistic interactions have been identified between these species, and PA readily outcompetes SA *in vitro* in mixed cultures (Machan et al., 1992; Duan et al., 2003; Mashburn et al., 2005; Park et al., 2012; Korgaonkar et al., 2013; Baldan et al., 2014; Fugère et al., 2014; Rüger et al., 2014; Filkins et al., 2015; Nguyen et al., 2015). We therefore wanted to determine if the two species could be stably maintained in our continuous-flow culture system. We found that a mixed species co-culture of PA and SA could be readily maintained to yield an apparently stable steady-state composition using Q = 170 μL min^–1^. The CFU counts for each species are shown in Figure 3A, and the co-culture OD_600_ measurements are shown in Supplementary Figure S2. A steady state composition of around 10^7^ SA CFU mL^–1^ and 10^8^ PA CFU mL^–1^ was established by 24 h of growth, and there were no significant differences in the viable cell counts following this (*P* > 0.05) up to 96 h of growth. By contrast, during aerobic batch culture in flasks, PA rapidly outcompeted SA and no viable SA could be recovered at the 96 h sampling point (Figure 3B). PA also outcompeted SA during stirred batch co-culture conditions (Q = 0 μL min^–1^) in the continuous-flow vessel (Figure 3C), albeit at a slower rate. Taken together, these data indicate that a continual supply of fresh media and removal of waste products is crucial for permitting a successful PA-SA co-culture *in vitro*.

#### Dual-Species Co-culture (PA-CA)

**FIGURE 4:**
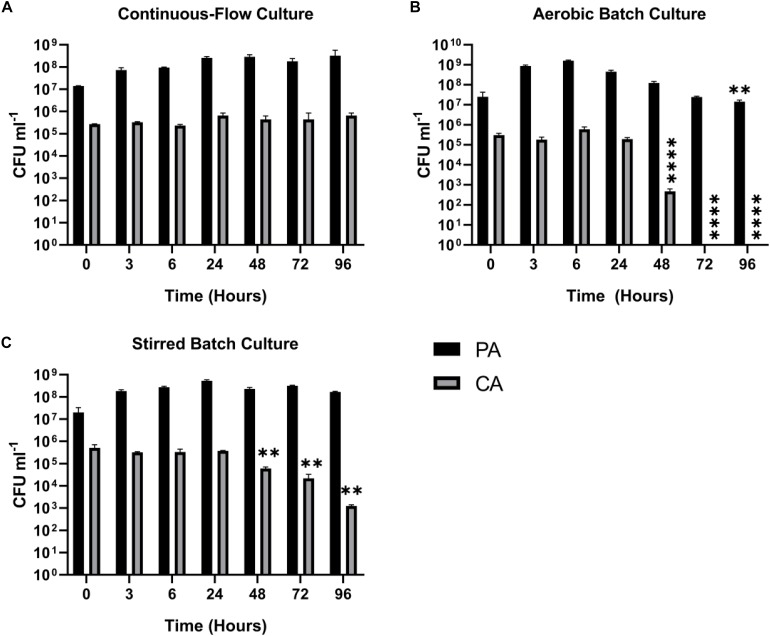
Continuous-flow culture allows *P. aeruginosa* and *C. albicans* to be maintained in a stable steady-state. Viable cell counts (CFU) of *P. aeruginosa* PAO1 (PA, black bars) and *C. albicans* SC5314 (CA, grey bars) during co-culture in ASM using: **(A)** a continuous-flow setup; **(B)** aerobic batch culture; and **(C)** stirred batch culture. Data represent as mean ± standard deviation from three independent experiments. Asterisks represent significant differences in CFU mL^– 1^ counts in comparison with the data from the 24 h time point (^∗∗^*P* < 0.005, ^∗∗∗∗^*P* < 0.0001).

Fungi, such as *Candida* sp. and *Aspergillus* sp. are also associated with CF airway infections (Williams et al., 2016; Bouchara et al., 2018). We therefore examined whether *Candida albicans* (CA) could be maintained alongside PA in the continuous-flow setup. This is important because inter-kingdom interactions between PA and CA have been previously shown to affect virulence factor production by both species (Hogan et al., 2004; McAlester et al., 2008; Cugini et al., 2010; Holcombe et al., 2010). We found that a co-culture of PA and CA could be readily maintained in the continuous-flow setup (Figure 4A), although to prevent a wash-out of CA from the culture vessel over time we had to decrease the flow rate (Q = 145 μl min^–1^). The culture carrying capacity for CA (ca. 10^5^ CFU mL^–1^) was lower than it was for PA (ca. 10^8^ CFU mL^–1^), but once a steady-state had been achieved (after 24 h incubation) no statistically significant differences in PA or CA viable cell counts were observed (*P* > 0.05). In contrast, CA titres rapidly declined during aerobic batch co-culture (Figure 4B). A similar, albeit slower decline in CA titres was observed during stirred batch growth (Figure 4C).

#### Dual-Species Co-culture (SA-CA)

**FIGURE 5:**
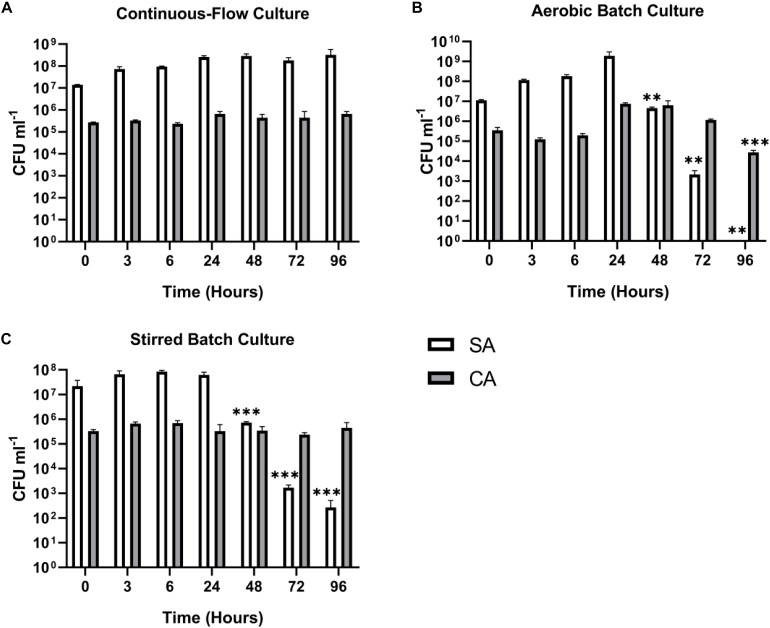
Continuous-flow culture allows *S. aureus* and *C. albicans* to be maintained in a stable steady-state. Viable cell counts of *S. aureus* 25923 (SA, white bars) and *C. albicans* SC5314 (CA, grey bars) co-cultures in ASM in: **(A)** a continuous-flow setup; **(B)** aerobic batch culture; and **(C)** stirred batch culture. Data represented as mean ± standard deviation of three independent experiments. CFU mL^– 1^ values are plotted on a log_10_ scale and asterisks represent significant differences in CFU mL^– 1^ counts in comparison with the data from the 24 h time point (^∗∗^*P* < 0.005, ^∗∗∗^*P* < 0.001, ^∗∗∗∗^*P* < 0.0001).

We next wanted to confirm that a stable co-culture of SA and CA could be maintained independent of PA. Using Q = 145 μL min^–1^, this was indeed the case (Figure 5A), and after 24 h growth, the ratio of SA:CA remained essentially unchanged. As in the PA-CA co-culture, at steady-state, the carrying capacity (ca. 10^5^ CFU mL^–1^) for CA was lower than it was for SA (ca. 10^8^ CFU mL^–1^). Unexpectedly, we noted that following aerobic and stirred batch culture, the CA outcompeted the SA (Figures 5B,C). This confirms that in mixed cultures, a species comprising just 0.1% of the microbiota can potentially have a major impact on titres of the [initially] numerically-dominant organism.

### Triple-Species Co-culture

**FIGURE 6:**
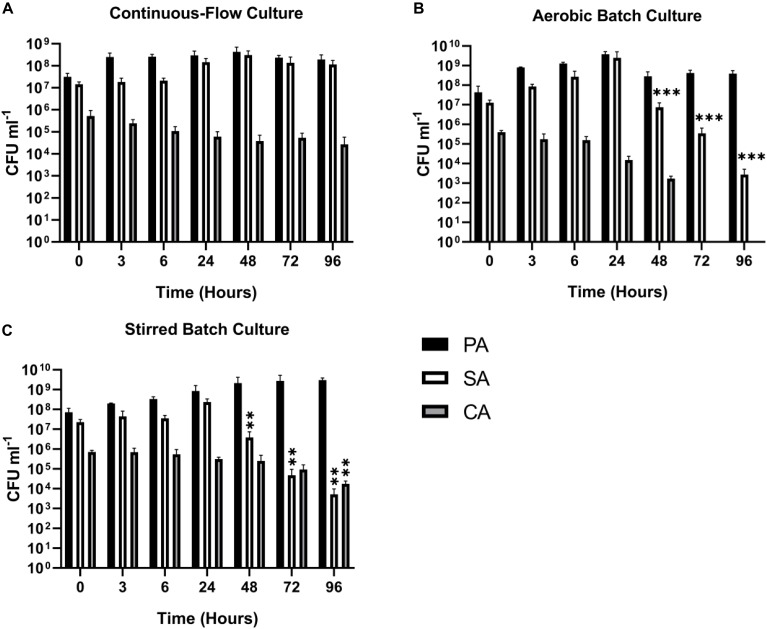
Continuous-flow culture allows *P. aeruginosa*, *C. albicans*, and *S. aureus* to be maintained in a stable steady-state. Viable cell counts of *P. aeruginosa* PAO1 (PA, black bars) *S. aureus* 25923 (SA, white bars) and *C. albicans* SC5314 (CA, grey bars) co-cultures in ASM in: **(A)** a continuous-flow setup; **(B)** aerobic batch culture; and **(C)** stirred batch culture. Data represented as mean ± standard deviation of three independent experiments. CFU mL^– 1^ values are plotted on a log_10_ scale and asterisks represent significant differences in CFU mL^– 1^ counts in comparison with the data from the 24 h time point (^∗∗^*P* < 0.05, ^∗∗∗^*P* < 0.001).

With the continuous-flow culture system clearly capable of maintaining dual-species co-cultures of PA-SA, PA-CA and CA-SA, we next wanted to determine if all three species could be co-cultured to achieve a stable steady-state composition. We found that setting Q = 145 μL min^–1^, a mixed population of all three microbial species could be maintained at a steady state for 96 h of incubation (Figure 6A; the corresponding in-line OD_600_ data are shown in Supplementary Figure S3). Once the steady-state had been achieved (i.e., after 24 h of growth) there were no significant differences in the CFU mL^–1^ counts for each species for the remaining duration of the co-culture (*P* > 0.1). The PA and SA titres remained at around 10^8^–10^9^ CFU mL^–1^, and the CA titres remained at around 10^5^ CFU mL^–1^. By contrast, when co-cultured in aerobic batch culture, both SA and CA were outcompeted by PA (Figure 6B). Indeed, there was a progressive decrease in the number of SA CFUs in each of the samples harvested after the 24 h time-point (*P* < 0.0005), and by 72 h, no viable CA CFU could be recovered. However, and unlike the PA-SA aerobic dual cultures (Figure 3B), SA could still be recovered at the 96 h sampling point, suggesting that the presence of CA affords a degree of protection, perhaps by decreasing the direct competition between PA and SA for shared resources. The stirred batch co-cultures yielded a somewhat different pattern (Figure 6C). Here, following the 24 h sampling point, PA titres remained high (ca. 10^9^–10^10^ CFU mL^–1^) and constant, but there was a significant and progressive decrease in SA titres (*P* < 0.05). Unlike the aerobic batch culture, this was accompanied by a much slower decline in CA titres.

### Quantification of *P. aeruginosa* Quorum Sensing Molecules

**FIGURE 7:**
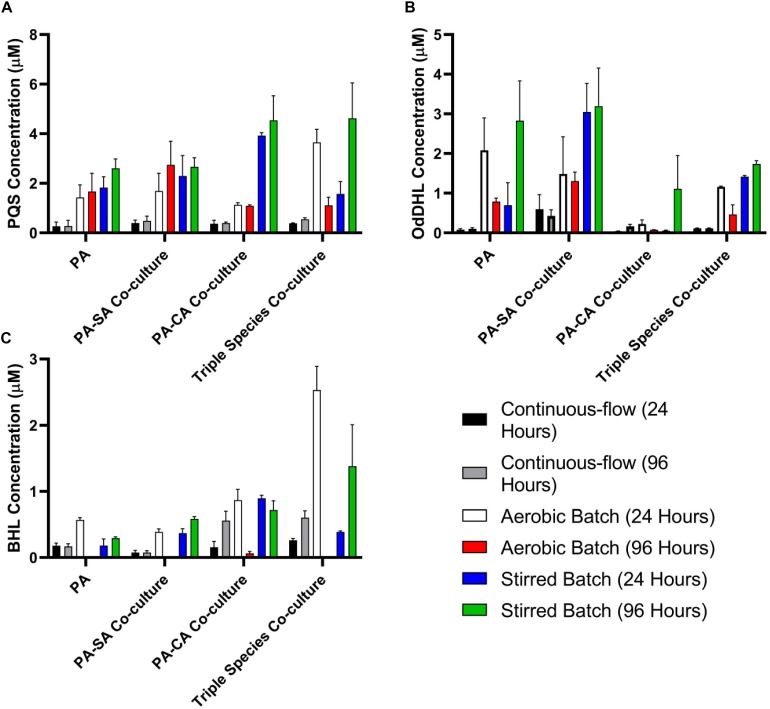
Quorum sensing molecule accumulation in the different culture setups. Concentration of the indicated *P. aeruginosa* quorum sensing molecules in the supernatant of single-species or multi-species co-cultures after 24 and 96 h incubation (as indicated). **(A)** Pseudomonas quinolone signal (PQS); **(B)** *N*-(3-oxododecanoyl)-L-homoserine lactone (OdDHL); **(C)** *N*-butanoyl-L-homoserine lactone (BHL). Data represented as mean ± standard deviation of three independent experiments.

Quorum sensing (QS) mediated signalling pathways are linked to the regulation of secondary metabolite and extracellular virulence factor production by PA. Some of these QS-regulated factors have been implicated in mediating interactions with other microbial species (Gambello et al., 1993; Smith and Iglewski, 2003; Lau et al., 2004; Schuster and Greenberg, 2006; Dekimpe and Déziel, 2009; Antunes et al., 2010). To examine how other microbial species might impinge on QS in PA, we therefore determined the concentration of the Pseudomonas quinolone signal (PQS), *N*-(3-Oxododecanoyl)-L-homoserine lactone (OdDHL) and *N*-butanoyl-L-homoserine lactone (BHL) in the culture supernatant of single and mixed species co-cultures (Figures 7A–C, respectively).

The concentration of all three QS molecules was significantly (*P* < 0.0001) lower in the continuous-flow setup compared with the aerobic- and stirred-batch cultures. In the continuous-flow setup, there was no significant difference in the concentration of PQS between the 24 and 96 h sampling points, or of OdDHL between these sampling points (*P* > 0.1), although we did note an increase in BHL concentration in the PA-CA co-culture over this period (*P* > 0.05). In contrast, QS molecules accrued to much higher concentrations in the aerobic- and stirred-batch cultures. Moreover, the presence of co-cultivated species had a large, but differential impact on QS molecule production by PA. For example, in batch culture, SA appeared to stimulate OdDHL production, whereas CA appeared to depress OdDHL levels and stimulate PQS (and to a lesser extent, also BHL) production. Taken together, our data indicate that QS molecules accumulate to a much lower concentration in the continuous-flow setup compared with batch cultures.

### Quantification of Pyocyanin

**FIGURE 8:**
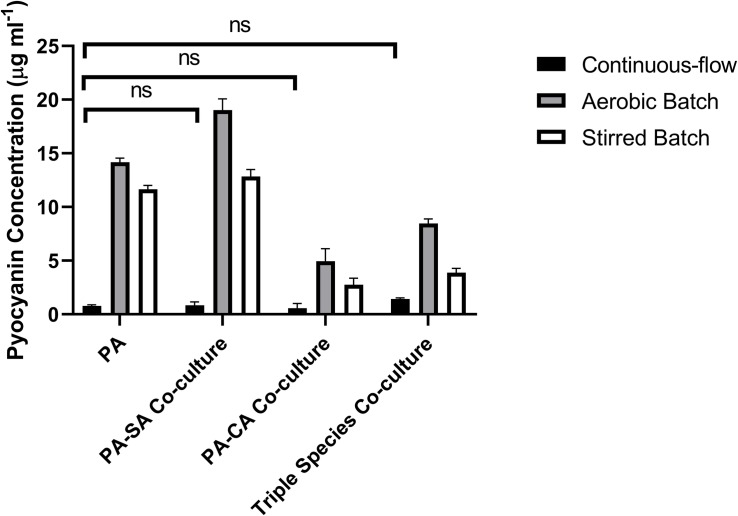
Pyocyanin accumulation in the different culture setups. The concentration (μg mL^– 1^) of pyocyanin in the supernatants of single-species and co-cultures after 96 h of incubation under: continuous-flow (black bars); aerobic batch (grey bars) and; stirred batch (white bars) culture conditions. Data represented as mean ± standard deviation of three independent experiments, *P* > 0.05 is considered no significant difference (ns).

Pyocyanin is a redox-active PA secondary metabolite, and is linked with virulence and competition between microbial species in the CF lung (Castric, 1975; Hoffman et al., 2006; Voggu et al., 2006; Biswas et al., 2009; Filkins et al., 2015; Noto et al., 2017). We measured pyocyanin levels in the different culture setups at the endpoint of each experiment (Figure 8). Pyocyanin concentrations were significantly lower for all microbial species combinations in the continuous-flow setup compared with the aerobic- or stirred-batch cultures (*P* < 0.0001). No significant differences were observed in pyocyanin accumulation between the different microbial co-culture combinations following growth in the continuous-flow setup (*P* > 0.3). However, we did note that in the batch cultures, the presence of CA depressed pyocyanin accumulation.

### Estimation of Mutation Rates in Co-cultures of *P. aeruginosa* and *S. aureus*

**FIGURE 9:**
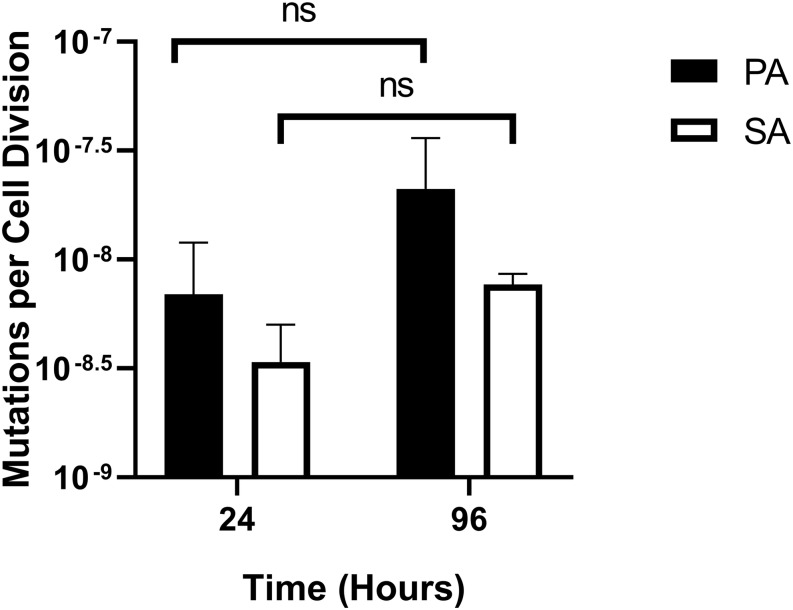
Mutation rates of *P. aeruginosa* and *S. aureus* during co-culture in the continuous-flow setup. Assumed mutation rates of *P. aeruginosa* PAO1 (black bars) and *S. aureus* 25923 (white bars) in the continuous-flow culture vessel after 24 and 96 h of incubation. Mutation rates were calculated as the number of Rif^R^-conferring mutations per cell per cell division, calculated as described by Foster (2006). The bars represent the mean ± standard deviation from three independent experiments. *P* > 0.05 is considered not significantly different (ns).

One possible use of the continuous-flow system described here would be to investigate how the presence of co-habiting species affects evolutionary trajectory(s). To gauge this, we measured the mutation rate of each species during co-culture. Mutation rates were measured as described by Foster (2006) and were assessed shortly after the steady-state had been attained (i.e., at the 24 h time-point) and at the end of the experiment (96 h time-point). The mean number of Rif^R^-conferring mutations per cell division was comparable with previously-reported values [≈10^–8^–10^–9^ mutations/cell/division (Schaaff et al., 2002; Dettman et al., 2016)] and was consistently low for both PA and SA, with no statistically significant differences between the 24 and 96 h sampling points (*P* > 0.1) (Figure 9). We conclude that PA and SA do not exhibit abnormal mutability in the continuous-flow setup and that co-culture of these species has no apparent impact on their respective mutation rate.

### Continuous-Flow Cultures Maintain a Constant pH

We also examined the endpoint pH of mixed-species cultures to see whether this differed from the starting pH of ASM (pH 6.7). We found that irrespective of the microbes and combinations of microbes being tested, the continuous flow cultures maintained a remarkably constant pH that was close to the starting pH. Stirred batch cultures maintained a pH of ca. 7, whereas aerobic batch cultures exhibited an endpoint pH > 8 (Supplementary Figure S4).

## Discussion

In this work, we have shown that a simple *in vitro* continuous-flow co-culture system enables long-term co-culture of three distinct microbial species (PA, SA and CA) associated with CF airway infections. When co-cultured in batch, these organisms ordinarily outcompete one another, leading to domination by a single species. However, in the setup described here, once a steady-state has been achieved (after around 24 h incubation) each inoculated species can be maintained at a constant titre, presumably reflecting the carrying capacity for each organism in the culture. Significantly, we show that even low-abundance species (represented by CA in our model) can be stably maintained, and that the presence of such species can have a major impact on the population trajectory of numerically more-abundant organisms such as SA, as well as inter-cellular signalling by PA.

The airways of people with CF have been shown to harbour a diverse polymicrobial community, comprising both bacteria and fungi (Sibley et al., 2006, 2008; Rogers et al., 2010a; Zhao et al., 2012; Carmody et al., 2013, 2015; Short et al., 2014; Boutin et al., 2015), and through the efforts of several teams, we now have a well-defined ASM for *in vitro* analyses. Indeed, PA grown in ASM has an almost identical gene expression profile compared with PA grown directly in sputum derived from CF patients (Turner et al., 2015). In spite of this, to date, there have been no reports describing the successful, long-term co-culture of CF-associated microbes in ASM. As we demonstrate in the current work, simply adding mixed-species inocula into ASM is not a recipe for the long-term maintenance of a stable population. Perhaps the best measure of the lack of progress on this front is seen when considering PA and SA. These two species are common in CF infections, and decades of work have revealed a wealth of knowledge about their physiology and nutritional requirements in axenic culture. However, until now, there have been no studies describing the successful long-term co-cultivation of these two species *in vitro*. One possible reason for this is that in iron limited conditions, PA lyses SA and uses the resulting lysate as a source of iron (Mashburn et al., 2005). By providing a continual supply of fresh media (which presumably mimics the unrelenting and exuberant production of airway secretions in the CF lung) we speculate that this nutritional limitation may be overcome.

The *in vitro* system described here offers a number of advantages. First, it is inexpensive to set up, making it accessible as a model to most researchers. Second, it is compliant with the “3Rs” (the replacement, refinement and reduction of animal research). Third, it is robust, as attested by the remarkably constant titres of each species following attainment of the steady-state condition. Fourth, early indications are that it can faithfully maintain species diversity when patient-derived CF sputum is being used to inoculate the system, and our progress on that aspect of the model will be published presently. Fifth, the system is far more defined and controlled than an animal model, allowing facile experimental perturbation. This experimental tractability means that we can address biological questions in a way that is just not possible with, e.g., animal models. For example, new species or defined mutants can be readily introduced to examine their impact on succession dynamics, and the action of antibiotics on the entire community can be accessed. We have also been exploring ways of modifying the setup to promote biofilm growth in the culture vessel, and again, these findings will be published in the near future.

Our *in vitro* setup is also subject to a number of perceived disadvantages. Unlike an animal model, it does not incorporate any immune response. This may be significant since the immune response would be expected to play a major role in clearance of microbes from the airways, and therefore exerts a selective pressure on the microbial community. Also, our model does not incorporate other types of host cell. This may be significant because in some circumstances (e.g., in patients carrying the DF508 CFTR mutation) the altered cell surface on the epithelia lining the airways has been implicated in promoting microbial colonisation (Campodónico et al., 2008). Mitigating these features, we note that few animal models accurately recapitulate the human CF airway environment, and aside from the difficulties associated with controlling and sampling such models, as far as we are aware, none of these models have yet been developed for maintaining a polymicrobial community of CF pathogens (O’Brien and Welch, 2019). One other potential disadvantage of our model is the requirement for continual flow. On the one hand, this is a feature that does allow maintenance of a stable steady-state community of microbes. On the other hand, even at low Q values, “washout” may prevent slow-growing species/variants from thriving, or key molecules from accumulating. For example, we noted that QS molecules (and pyocyanin) fail to accumulate in the continuous-flow system, whereas these compounds reached high levels in batch culture. The most likely explanation for this is simple washout (through continual dilution) of the QS signals. However, it should be noted that with Q = 170 μL min^–1^, it would take > 6 h to dilute the vessel contents by 50%, and all the while, the contained culture continues to grow and elaborate more QS molecules. To put this into context, previous work has shown that QS molecules more than double their concentration in batch cultures in a 2 h period (Davenport et al., 2015), so assuming similar kinetics in ASM, these molecules should accumulate faster than they are diluted. If so, this suggests that QS plays a less important role in continuous-flow cultures than it does in batch cultures. The low steady-state concentrations of QS molecules documented here may also be advantageous (for the experimenter). First of all, the metabolic physiology of the community is defined and stable over the experiment’s time course and we would not expect to see the bursts of metabolic activity which would normally accompany the accumulation of QS molecules in the post-quorate period (Davenport et al., 2015). Second, and if the effect(s) of QS molecules on community interactions does need to be examined, this can be easily be done through the addition of defined concentrations of exogenous QS molecules.

We conclude that the setup described here enables facile maintenance of PA, SA and CA (a Gram-negative bacterial species, a Gram-positive bacterial species and dimorphic fungus, respectively). Our approach provides a framework for potentially recapitulating the entire polymicrobial community associated with CF airway infections. The setup will provide leverage to access to key biological problems regarding inter-species interactions, the impact of antibiotics, and the impact that newly-introduced species may have on the community trajectory.

## Data Availability Statement

The raw data supporting the conclusions of this manuscript will be made available by the authors, without undue reservation, to any qualified researcher.

## Author Contributions

TO’B and MW conceived and designed the work and revised the manuscript. TO’B executed the experiments, analysed the data, and drafted the manuscript.

## Conflict of Interest

The authors declare that the research was conducted in the absence of any commercial or financial relationships that could be construed as a potential conflict of interest.
