# Supplementary material for: A Continuous-Flow Model for in vitro Cultivation of Mixed Microbial Populations Associated With Cystic Fibrosis Airway Infections
Source: Front Microbiol. 2019 Nov 22;10:2713. doi: 10.3389/fmicb.2019.02713 (PMC6883238; doi:10.3389/fmicb.2019.02713)
Supplement: Supplementary file 1 [file Data_Sheet_1.docx]

**Supplementary Information**

**Preparation of artificial sputum media (ASM)**

The following protocol was used to prepare 1 L of ASM, this modified recipe is a combination of previously published protocols for the production of SCFM2 (Palmer *et al*. (2007)(1) and Turner *et al*. (2015)(2)) and ASM (Kirchner *et al*. (2012)(3)).

1. Add 5 g of mucin from porcine stomach, type-II (Sigma-Aldrich) to 250 mL of sterile 1 x PBS (Oxoid) (final concentration = 1.25 g L^-1^) and leave to dissolve with stirring overnight at 4°C.
2. Add 4 g of fish sperm DNA (Sigma-Aldrich) to 250 mL of sterile distilled H_2_O (dH_2_O) (final concentration = 1 g L^-1^) and leave to dissolve overnight in a shaking water bath at 35°C, 180 rpm.
3. In a clean beaker, add 250 mL of dH_2_O and make up the SCFM2 buffered base and amino acid mix using the recipe below (Table S1).

| **Buffered Base stocks** | | | | | | | |
| --- | --- | --- | --- | --- | --- | --- | --- |
| Chemical | [Stock] | stock volume | fw | Mass to add | Stock to add to beaker | Final Conc | Notes |
|  | (M) | (mL) | (g/mol) | (g) | (mL) | (mM) | - |
| NaH_2_PO_4_ | 0.2 | 25 | 137.99 | 0.690 | 8.125 | 1.3 | - |
| Na_2_HPO_4_ | 0.2 | 25 | 141.96 | 0.710 | 6.252 | 1.25 | - |
| KNO_3_ | 1 | 25 | 101.103 | 2.528 | 0.348 | 0.348 | - |
| K_2_SO_4_ | 0.25 | 25 | 174.259 | 1.089 | 1.084 | 0.271 | - |
| **Add solids below directly to beaker** | | | | | | | |
| NH_4_Cl |  |  | 53.491 | 0.124 |  | 2.2808 | - |
| KCl |  |  | 74.5513 | 1.116 |  | 14.943 | - |
| NaCl |  |  | 58.44 | 3.032 |  | 51.848 | - |
| MOPS |  |  | 209.2633 | 2.092 |  | 10 | - |
| **Amino Acids** | | | | | | | |
| Ser | 0.1 | 50 | 105.09 | 0.525 | 14.46 | 1.446 | - |
| Glu.HCl | 0.1 | 50 | 183.59 | 0.918 | 15.492 | 1.549 | - |
| Pro | 0.1 | 50 | 115.13 | 0.576 | 16.612 | 1.661 | - |
| Gly | 0.1 | 50 | 75.07 | 0.375 | 12.032 | 1.203 | - |
| Ala | 0.1 | 50 | 89.09 | 0.445 | 17.8 | 1.78 | - |
| Val | 0.1 | 50 | 117.15 | 0.586 | 11.172 | 1.117 | - |
| Met | 0.1 | 50 | 149.21 | 0.746 | 6.332 | 0.633 | - |
| Ile | 0.1 | 50 | 131.17 | 0.656 | 11.212 | 1.121 | - |
| Leu | 0.1 | 50 | 131.17 | 0.656 | 16.092 | 1.609 | - |
| Orn.HCl | 0.1 | 50 | 168.62 | 0.843 | 6.76 | 0.676 | - |
| Lys.HCl | 0.1 | 50 | 182.6 | 0.913 | 21.28 | 2.128 | - |
| Arg.HCl | 0.1 | 50 | 210.7 | 1.054 | 3.06 | 0.306 | - |
| Trp | 0.1 | 50 | 204.23 | 1.021 | 0.132 | 0.013 | Prep in 0.2 M NaOH |
| Asp | 0.1 | 50 | 133.1 | 0.666 | 8.272 | 0.827 | Prep in 0.5 M NaOH |
| ^*^Tyr | 0.1 | 50 | 181.19 | 0.906 | 8.02 | 0.802 | Prep in 1.0 M NaOH |
| ^*^Thr | 0.1 | 50 | 119.12 | 0.596 | 10.72 | 1.072 | - |
| ^*^Cys.HCl | 0.1 | 50 | 157.6 | 0.788 | 1.6 | 0.16 | - |
| ^*^Phe | 0.1 | 50 | 165.19 | 0.826 | 5.3 | 0.53 | - |
| ^*^His.HCl.H_2_O | 0.1 | 50 | 209.6 | 1.048 | 5.192 | 0.519 | - |

**Table S1** Instructions for the preparation of stock solutions required to make the buffered base and amino acid mixture. The amount of each solution required to make 1 L of ASM is shown alongside the concentration (mM) of each constituent component within the culture media. Note that all amino acid stock solutions can be kept in the dark at 4°C for 1 month, stock solutions annotated with ‘*’ must be made fresh on the day of preparation.

1. Combine the dissolved mucin and DNA solutions and pellet undissolved particles by centrifugation at 4000 × *g* for 30 min (4°C).
2. Without disturbing the pellet, carefully remove the supernatant and combine with the buffered base-amino acid mix.
3. Adjust the pH of the media to 6.8.
4. Add the final components of ASM as shown in Table S2.

| Chemical | [Stock] | Stock volume | fw | Mass to add | Stock to add to 1L mL BB | [Final] | Notes |
| --- | --- | --- | --- | --- | --- | --- | --- |
|  | (M) | (mL) | (g/mol) | (g) | (mL) | (mM) |  |
| Dextrose (D-glucose) | 1 | 25 | 180.16 | 4.504 | 1.2 | 3 |  |
| L-lactic acid | 1 | 25 | 90.08 | 2.252 | 9.3 | 9.3 | pH stock to 7 with NaOH |
| CaCl_2_.2H_2_O | 1 | 25 | 147.014 | 3.67535 | 1.754 | 1.754 |  |
| MgCl_2_.6H_2_O | 1 | 25 | 203.31 | 5.08275 | 0.606 | 0.606 |  |
| FeSO_4_.7H_2_O | 0.0036 | 50 | 278.05 | 0.05 | 1 | 0.0036 | Make fresh on day of preparation |
| *N*-acetylglucosamine | 0.25 | 25 | 221.21 | 1.383 | 1.2 | 0.3 |  |
| Egg yolk emulsion |  | | | | 5mL |  | Premade stock from Sigma |

**Table S2**. Instructions for the preparation of stock solutions of the final reagents required to make ASM. The amount of each stock solution required to make 1 L ASM is shown alongside the final concentration (mM) of each constituent component within the culture media. Note that the FeSO_4_ stock must be prepared fresh on the day of preparation.

1. Adjust the final volume to 1 L with dH_2_O.
2. Filter sterilise media using a 0.22 µm Millipore stericup (Sigma-Aldrich) attached to a vacuum pump. Note that this process can be slow and may take up to two days.
3. Store filtered ASM in the dark at 4°C for up to one month.


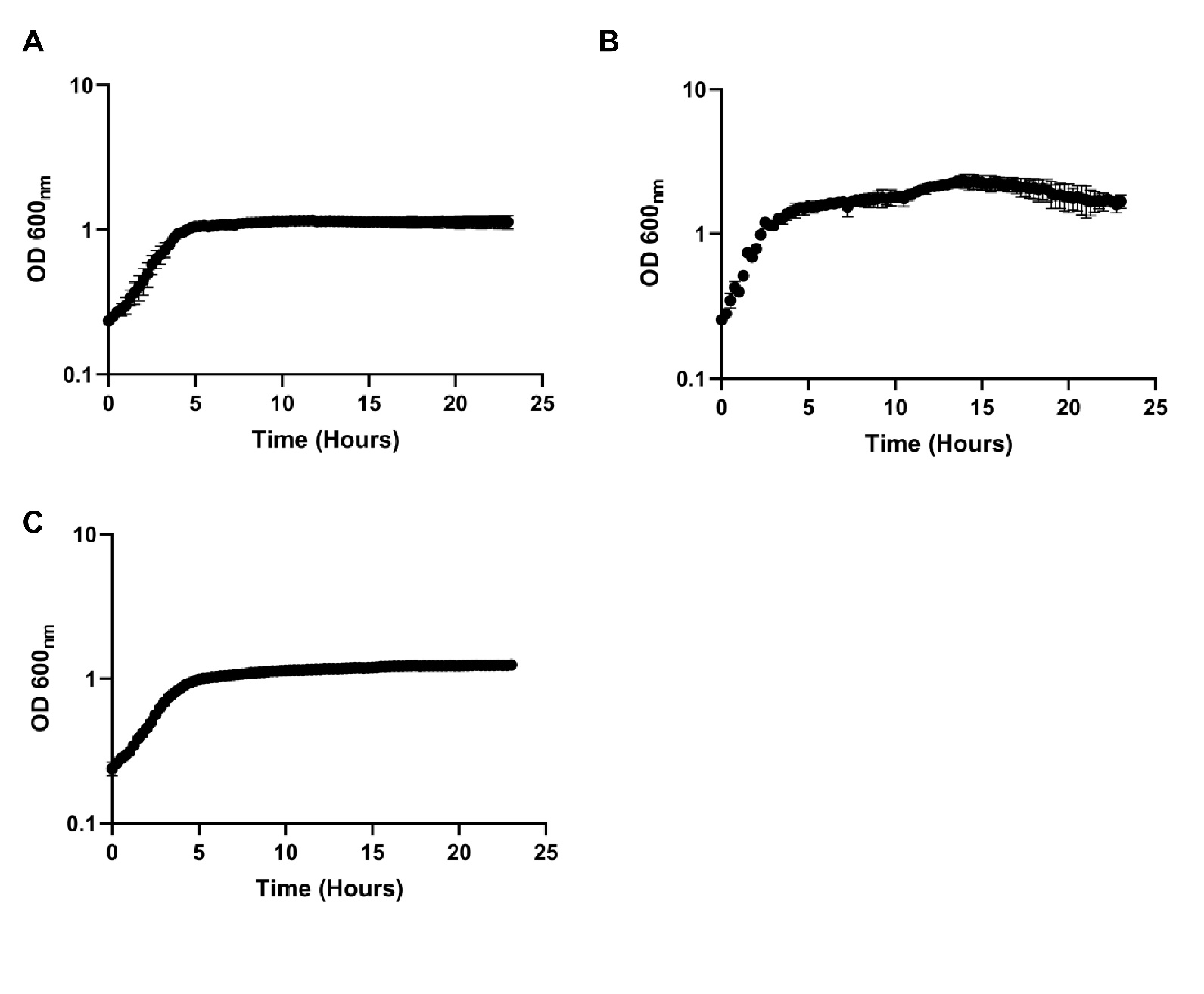


**Figure S1.** Growth of **(A)** *Pseudomonas aeruginosa* PAO1; **(B)** *Staphylococcus aureus* 25923; **(C)** *Candida albicans* SC5314 in ASM. All cultures were grown within flat-bottomed 96 well microtiter plates (Nunc) incubated at 37°C. Measurements were taken every 15 mins using a FluoStar Omega plate reader (BGM) with 180 orbital shaking during idle time between reads. Optical density at 600 nm is plotted on a log­_10_ scale with data presented as the mean ± standard deviation from at least 3 independent experiments.


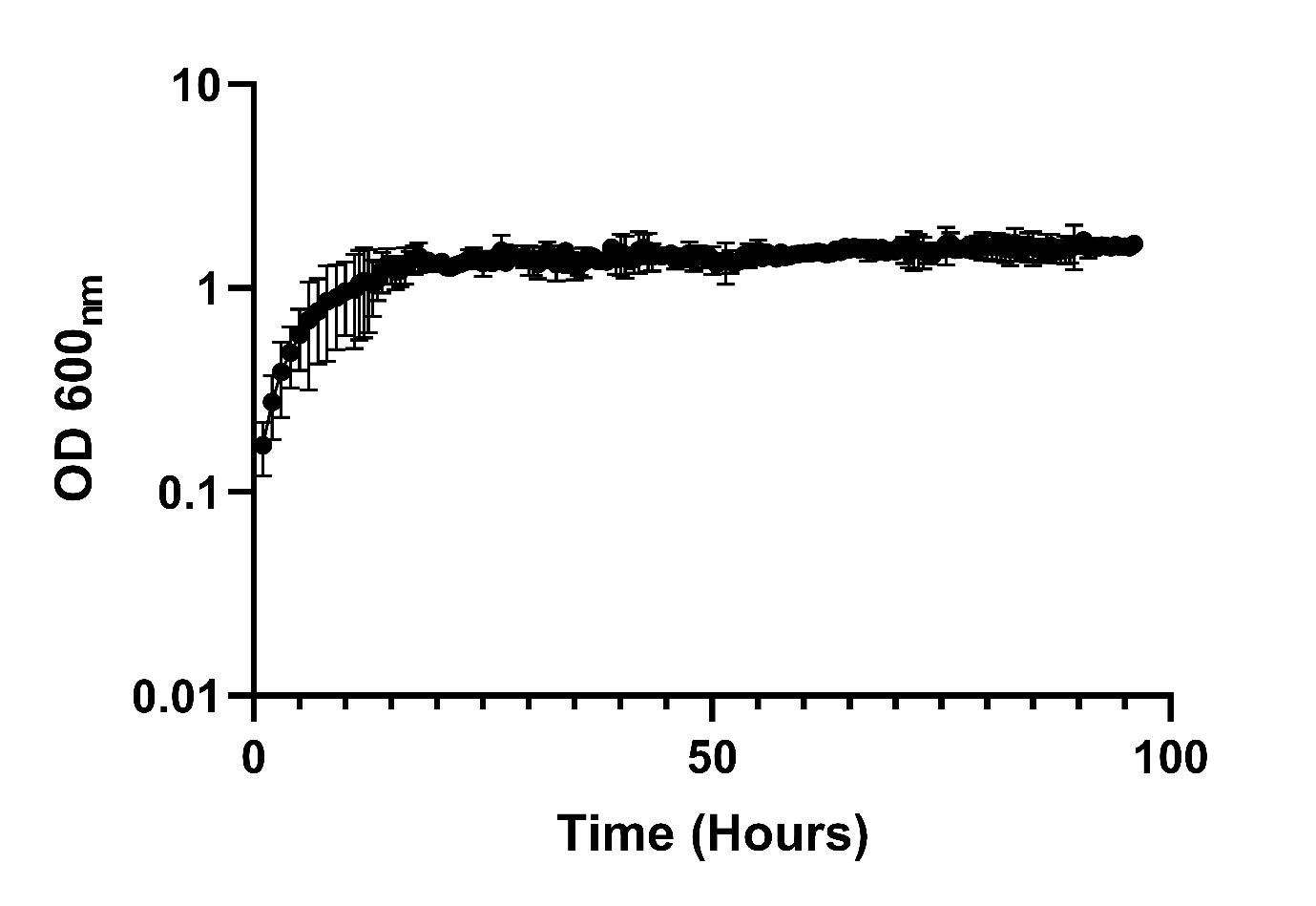


**Figure S2.** Optical density at 600 nm of a *Pseudomonas aeruginosa* PAO1 - *S. aureus* 25923 co-culture in ASM within the continuous-flow culture vessel. Automated measurements were taken every 30 min by a 6715 UV series spectrophotometer (Jenway) fitted with a continuous-flow cuvette. Data represent the mean ± standard deviation from 3 independent experiments.


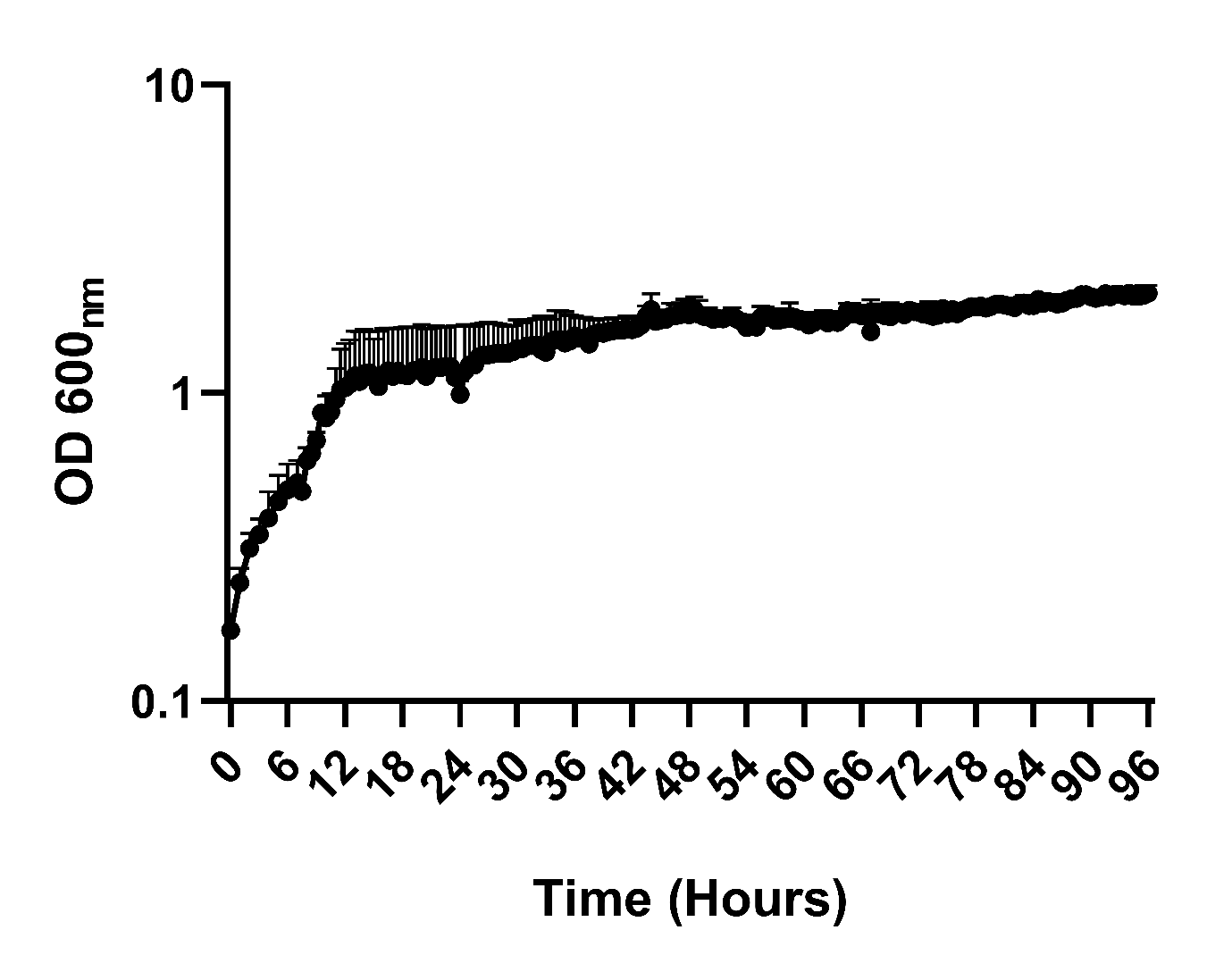


**Figure S3.** Optical density at 600 nm of *Pseudomonas aeruginosa* PAO1, *S. aureus* 25923 and *C. albicans* SC5314 co-culture grown in ASM in the continuous-flow system. Automated measurements were taken every 30 min using a 6715 UV series spectrophotometer (Jenway) fitted with a continuous-flow cuvette. Data represent the mean ± standard deviation from 3 independent experiments.

**Endpoint pH of Co-cultures**

Endpoint (96 hr) pH measurements in the continuous-flow system were significantly lower (P < 0.0001) for all microbial species combinations, compared with aerobic or stirred batch cultures. The average endpoint pH in the continuous-flow cultures was 6.5, which is comparable to the pH of pre-warmed, sterile ASM (~6.7). When quantifying the concentrations of BHL and OdDHL it was noted that in aerobic batch cultures, and to some extent, also stirred batch cultures, there was a lower concentration of these *N*-acyl homoserine lactones at the 96 hr sampling point. This may have been due to the increased pH of the culture media in batch culture conditions leading to hydrolysis of the lactone ring, as described by Gómez-Bombarelli *et al*. (2013)(4).

**
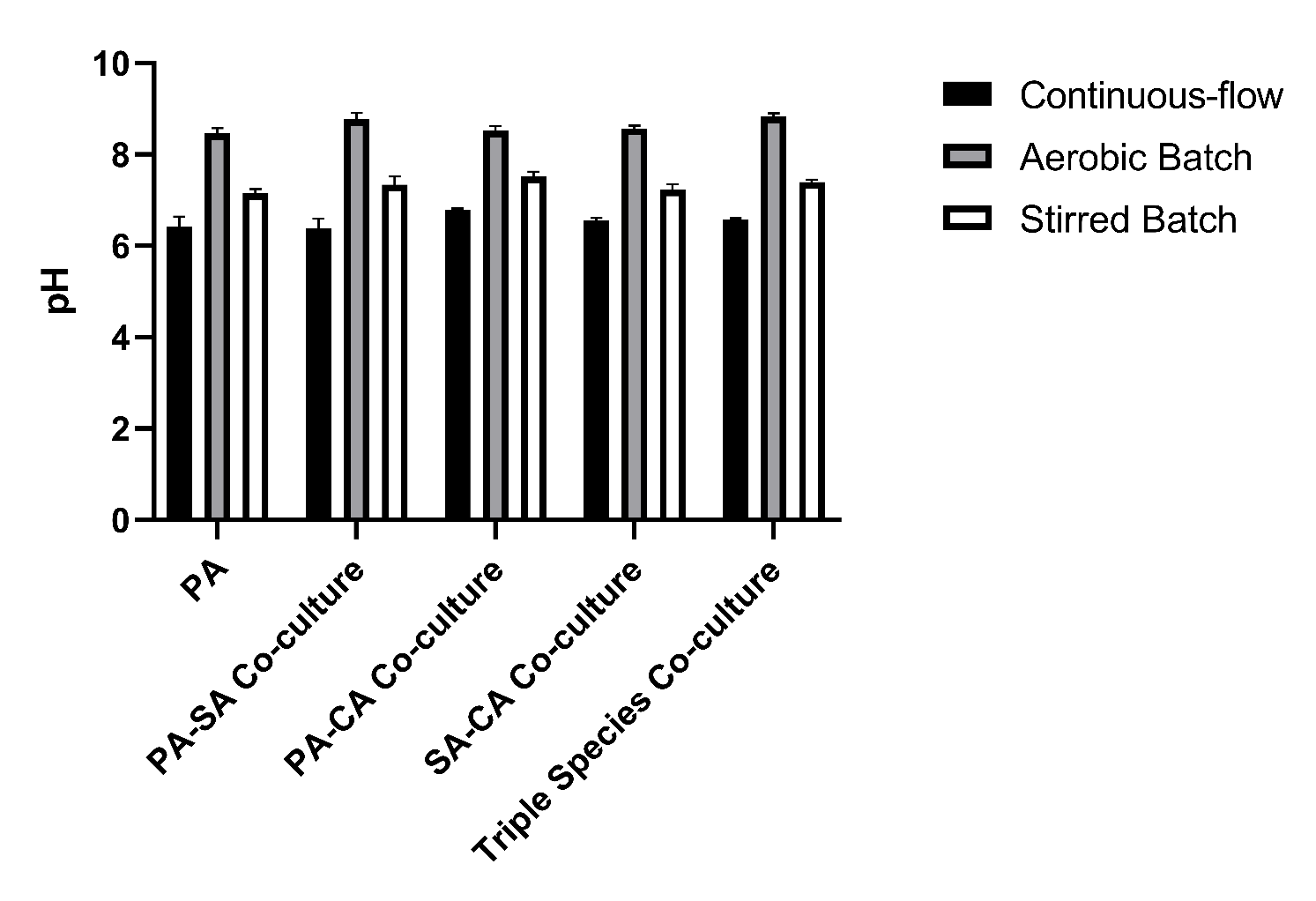
**

**Figure S4.** Endpoint pH measurements of single-species and co-cultures after 96 hr of incubation under: continuous-flow (black bars); aerobic batch (grey bars); and stirred batch (white bars) culture conditions. Data represented as mean ± standard deviation of three independent experiments.

**References**

1. K. L. Palmer, L. M. Aye and M. Whiteley: Nutritional cues control Pseudomonas aeruginosa multicellular behavior in cystic fibrosis sputum. *J Bacteriol*, 189(22), 8079-87 (2007) doi:10.1128/JB.01138-07

2. K. H. Turner, A. K. Wessel, G. C. Palmer, J. L. Murray and M. Whiteley: Essential genome of Pseudomonas aeruginosa in cystic fibrosis sputum. *Proc Natl Acad Sci U S A*, 112(13), 4110-5 (2015) doi:10.1073/pnas.1419677112

3. S. Kirchner, J. L. Fothergill, E. A. Wright, C. E. James, E. Mowat and C. Winstanley: Use of artificial sputum medium to test antibiotic efficacy against Pseudomonas aeruginosa in conditions more relevant to the cystic fibrosis lung. *J Vis Exp*(64), e3857 (2012) doi:10.3791/3857

4. R. Gómez-Bombarelli, E. Calle and J. Casado: Mechanisms of lactone hydrolysis in neutral and alkaline conditions. *J Org Chem*, 78(14), 6868-79 (2013) doi:10.1021/jo400258w
